# Severe Pulmonary Blastomycosis in a Young Adult: Probable Role of E-Cigarette Use in Immunosuppression

**DOI:** 10.1155/carm/6019638

**Published:** 2025-03-25

**Authors:** Rajat Gupta, Barath Prashanth Sivasubramanian, Ajay Sriram Antony Raj, Sudha Dirisanala, Tahani Dakkak, Ashley Williams, Navneeth Bongu

**Affiliations:** ^1^GME Department of Internal Medicine, Northeast Georgia Medical Center, Gainesville, Georgia, USA; ^2^Department of Medicine, Madras Medical College, Chennai, Tamil Nadu, India; ^3^GME Department of Research, Northeast Georgia Medical Center, Gainesville, Georgia, USA; ^4^Department of Pulmonary and Critical Care, Northeast Georgia Medical Center, Gainesville, Georgia, USA

**Keywords:** blastomycosis, e-cigarettes, pneumonia, respiratory health

## Abstract

**Background:** E-cigarette use has become increasingly prevalent among young adults, raising concerns about its potential health impact and susceptibility to infections. *Blastomyces dematitidis*, the causative agent of blastomycosis, is a dimorphic fungus endemic to certain regions of the United States. We present a rare presentation of pulmonary blastomycosis in a young, immunocompetent male with chronic e-cigarette use, highlighting the need to consider fungal infections in the differential diagnosis of pneumonia of unknown etiology in patients with risk factors for respiratory illness.

**Presentation:** A 20-year-old male with a 4-year history of daily e-cigarette use and gutter cleaner by occupation presented with a 10-day history of worsening cough, bloody sputum, shortness of breath, night sweats, fever, and weight loss. He was hemodynamically stable, required minimal oxygen support, and was admitted for chest X-ray showing right upper lobe pneumonia and cavitary lesion. The patient was tested for community-acquired pneumonia, atypical pneumonia, granulomatous diseases, and immunosuppressive and viral lymphoproliferative disorders. A diagnostic bronchoscopy ultimately confirmed blastomycosis, leading to treatment with amphotericin B and itraconazole, resulting in significant clinical improvement.

**Conclusion:** Pulmonary blastomycosis often presents asymptomatically, with severe cases more common in the elderly or those with comorbidities and immune dysfunction. Physicians tend to overlook it as a differential diagnosis for primary lung infections, focusing on bacterial pneumonia and tuberculosis in younger individuals. This case underscores the need for further investigation into the impact of e-cigarette use on immune function. This case also highlights the importance of making blastomycosis a reportable disease in Georgia, considering its increasing incidence and the widespread construction and soil disturbance occurring throughout the state.

## 1. Introduction

The modern e-cigarette, a battery-powered nicotine delivery device, was introduced in 2003 to reduce tobacco-related diseases [1]. By 2007, e-cigarettes entered the international market and quickly gained popularity, particularly among teenagers and young adults, due to their wide variety of flavors and appealing designs [2, 3]. Since e-cigarettes are a recent product largely promoted by the nicotine industry, the long-term health implications remain largely unknown [4]. Research suggests that vaping results in altered functioning of innate defense proteins in airway secretions, similar to cigarette smoking [5]. Most of the vaping-related infections discussed in the literature have either a bacterial or viral etiology [5–7]. There are very limited cases of fungal infections.

*Blastomyces dermatitidis* is a dimorphic fungus that exists as a mold in the environment (22°C–25°C) and as a yeast in the human body (37°C). It primarily causes a lung infection but can present with varying degrees of severity, from an asymptomatic infection to ARDS [8]. *Blastomyces* is endemic to the midwestern, south-central, and southeastern United States, typically found in forested, sandy soils near acidic water sources that contain decaying vegetation and organic material. Clinically and radiologically, blastomycosis often resembles other pulmonary conditions such as tuberculosis, lung cancer, or bacterial pneumonia [9]. In the absence of skin lesions, known risk factors, or a lack of response to antibiotics, blastomycosis is typically not considered in the list of differential diagnoses for patients presenting with these symptoms [9].

In this case report, we present a young adult with a history of chronic e-cigarette use diagnosed with pulmonary blastomycosis. The patient's young age, lack of underlying health conditions, and ambiguity in clinical presentation, along with ambiguous imaging findings, posed a significant diagnostic challenge.

## 2. Case

**Figure 1:**
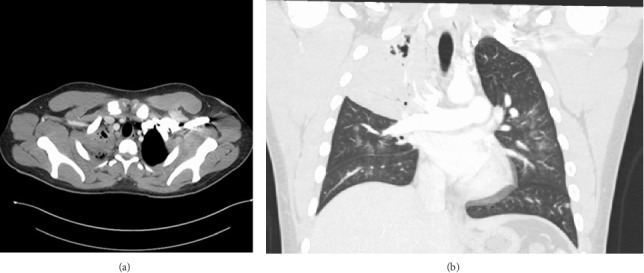
CT chest imaging. Consolidative opacities with cavitation seen throughout the right upper lobe in (a) the axial section and (b) the coronal section.

**Figure 2:**
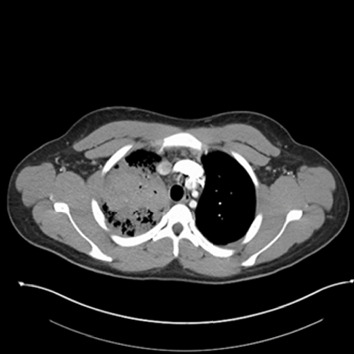
CT chest imaging. Patchy ground-glass consolidations were also seen in the right lower lobe. Mild centrilobular ground-glass opacities were present in the right middle lobe and lingula.

A 20-year-old male without significant past medical history presented to the emergency department (ED) with undocumented fever, productive cough with green–yellow sputum for the last 10 days, and 1 episode of small quantity hemoptysis that brought him to the ED. He also had mild shortness of breath at rest, night sweats, 12-pound weight loss, and pleuritic chest pain that began in the last 1 month. The patient resides in Georgia and has worked as a gutter installer and cleaner. The patient does not use a face mask while working, has a history of vaping nicotine daily for the last 4 years, and has 4 dogs at home. In the ED, his vital signs included a fever of 101.8 F, pulse of 108 beats per minute, respiratory rate of 23 per minute, and saturation at 96% on 3 L O_2_. On examination, crackles were audible in the right lower lobes and the remaining examination was unremarkable. Labs were pertinent for leukocytosis at 25,400 cells/mm^3^ and elevated CRP at 6.83 IU/L. The laboratory investigations are depicted in Table 1. Chest X-ray showed right upper lobe consolidation and CT chest was suggestive of small right pleural effusion and patch alveolar opacities throughout the right lower lobe. Pulmonology was consulted, and the patient was started on IV vancomycin, piperacillin/tazobactam, and azithromycin for acute respiratory failure secondary to presumable community-acquired pneumonia (CAP) with broad spectrum coverage including coverage for *Pseudomonas* with aim togradually transition to narrow spectrum antibiotcs as per the sensitivity reports. Subsequent CT chest with IV contrast 5 days later was suggestive of consolidative opacities in RUL with cavitation and ground-glass consolidation in RLL but no lymphadenopathy was noted. CT images from the subsequent scan are shown in Figures 1 and 2. The patient's test results are depicted in Table 1. He continued to spike fevers during the second and third days of hospitalization, and his WBC count rose to 35.5 and later to 41,800. His antibiotics were switched to meropenem and linezolid. Due to unresolved symptoms, the patient was diagnosed with CAP of unknown etiology, a bronchoscopy, RUL bronchoalveolar lavage, and 10-R fine-needle biopsy of the lymph node, and the RUL tissue were sent for further testing. BAL cytology showed predominantly neutrophils with an elevated CD4: CD8 ratio of 7 : 1 but was overall nonspecific.

The patient developed acute kidney injury secondary to ATN due to vancomycin and piperacillin/tazobactam and decreased oral intake with creatinine peaking at 3.13. However, it resolved within the next 4 days. His urine blastomycosis and histoplasmosis antigens came out to be positive. The tissue from RUL, taken during bronchoscopy, grew rare budding yeast organisms, the stain was consistent with blastomycosis, and later cultures also grew blastomycosis. However, the lymph node did not show any growth/cells. Antibiotics were changed to antifungals to cover moderate to severe blastomycosis pneumonia with amphotericin B 3 mg/kg. Zyvox and meropenem were discontinued. This led to the immediate resolution of fever, and WBC showed a downtrend. He did not require ICU admission or vasopressor support but did require oxygen via nasal cannula with the highest oxygen at 9L for one day followed by successful weaning off the oxygen. He was on amphotericin B for 1 week, followed by oral itraconazole at 200 mg three times daily for 3 days. His renal and liver functions did rise after being initiated on antifungals but they trended back to baseline on discharge, as shown in Table 1. The patient clinically improved, became hemodynamically stable, and was discharged on a reduced dose of itraconazole 200 mg twice daily. On 2 weeks outpatient follow-up, the patient had a mild cough with occasional productivity, nausea, and dyspnea on exertion. However, he noted an improvement in appetite and denied fevers. He was advised to continue oral itraconazole for at least 6 months and was cleared to return to work with the use of a high-filter mask. A follow-up CT scan was scheduled for 3 months postdischarge, but the patient subsequently transferred his care to another state.

## 3. Discussion

Blastomycosis is asymptomatic in most individuals and presents with severe disease only in aged patients or those who have comorbidities or immune derangements [10]. We were able to diagnose him appropriately by following the CDC guidelines on approaching patients with CAP of unknown etiology [11]. Blastomycosis is often not a top differential, particularly in the case of a primary lung infection. Bacterial pneumonia or tuberculosis is mostly the first suspected differential, as in this case [8]. *Blastomyces* spp. is commonly found in the midwestern, southeastern, and south-central United States, particularly around the Mississippi and Ohio River basins [12]. Our patient resides in Georgia, and this places him at higher risk for blastomycosis infection. Although Georgia lies in the blastomycosis endemic area, it is not a reportable disease yet [13]. The incidence of dimorphic fungi such as blastomycosis has been steadily on the rise and previously known epidemiologic patterns are changing. These changes are multifactorial with climatic change and anthropogenic activities hypothesized as leading factors [14]. Previous studies, including a 10-year trend analysis of United States' hospitalized patients with blastomycosis, showed that individuals over the age of 50 were the most frequently affected [15, 16]. In contrast, our patient was only 20 years old. Hospitalization among younger adults, as seen in this case, was uncommon [15, 16]. Approximately 50% of blastomycosis cases are asymptomatic, with severe disease commonly occurring in individuals with immunosuppression or underlying health conditions [10, 17]. Cari et al. observed that obesity, diabetes mellitus, and immunosuppression were risk factors associated with severe blastomycosis [18]. However, this patient was young and did not have any comorbidities adding to the uniqueness of the case. *Blastomyces dermatitidis* thrives in moist soil and decaying organic matter, making workers who disturb soil or vegetation susceptible to inhaling infectious spores [19]. Ireland et al. observed that more than three-fourths of patients with blastomycosis infection had engaged in soil-exposing or vegetation-related activities such as gardening, gathering wild plants, woodcutting, or being near construction or excavation work [20]. Our patient was a gutter cleaner putting him at an increased risk of exposure to the illness. Although findings from our case suggest that the patient's occupational exposure was likely the primary source of infection, we hypothesize that his chronic use of e-cigarettes in the absence of other comorbidities may have exacerbated the severity of his condition. People with impaired immunity are at an increased risk of developing more serious forms of blastomycosis infection [21]. Based on the laboratory findings, our patient exhibited no significant discrepancies in cellular or humoral immunity besides leukocytosis. Tommasi et al. showed that e-cigarette usage is associated with the downregulation of multiple immunoregulatory genes, especially in chronic users [22]. The most affected is the macrophage “C-C motif chemokine receptor 5” (CCR5) signaling, which controls how leukocytes, such as macrophages and T cells, move to areas of inflammation [22, 23]. The CSF-1 gene, the primary regulator of mononuclear phagocyte survival, proliferation, differentiation, and function, is also downregulated in e-cigarette users [24, 25]. A dysregulated immune system or impaired neutrophil, T cell, and macrophage activity means reduced immunological activity against fungal pathogens. This allows *Blastomyces* fungal spores to survive, transition, and replicate in alveolar macrophages, often causing early infection [10, 26]. Aside from infections, vaping has also been observed to cause asthma and COPD exacerbation and increase atherosclerotic damage, leading to myocardial infarction and stroke at a younger age [27–29]. Xie et al. also observed that current or former users of e-cigarettes had a higher risk of acquiring any kind of respiratory illness, in particular, chronic bronchitis, emphysema, asthma, and COPD [30]. E-cigarettes also affect the normal microbiota in the oral cavity [31].

Consolidation is the most common radiological feature of pulmonary blastomycosis, while cavitary lesions are relatively rare and occur less frequently than in histoplasmosis [32]. However, in contrast, Sheflin et al. reported that approximately one-third of patients with blastomycosis presented with cavitary lesions. It is challenging to distinguish between blastomycosis, tuberculosis, or bacterial pneumonia based on the radiological findings alone [33]. In our case, imaging revealed right upper lobe consolidation and cavitary lesions. In endemic regions, upper lobe infiltrates (airspace or mass-like) should prompt consideration of pulmonary blastomycosis [27]. For cases of CAP of unknown etiology that are unresponsive to empiric antibiotics, CAP patients with skin lesions, or those associated with a blastomycosis outbreak, the CDC's latest update on the testing algorithm for blastomycosis recommends starting with the enzyme immunoassay (EIA) urine antigen test [11]. In our case, bronchoalveolar lavage revealed fungal elements, prompting further testing of the urine for fungal antigens. Urinary fungal antigen testing turned out to be positive for both blastomycosis and histoplasmosis. Cross-reactivity among urinary antigens of *Blastomyces dermatitidis* and *Histoplasma capsulatum* occurs commonly [34]. Caution is advised when interpreting test results, as it does not necessarily imply multiple infections [34]. However, since the antifungal therapy options and treatment decisions are similar for both infections, urinary antigen assays are still useful for rapid diagnosis despite their cross-reactivity [34]. In our patient, *Blastomyces* were identified from bronchoalveolar lavage cultures, thereby confirming the diagnosis of pulmonary blastomycosis. The current recommendation for treating moderately severe to severe pulmonary blastomycosis is amphotericin B followed by oral itraconazole. This approach followed the 2008 update of the “Clinical Practice Guidelines for the Management of Blastomycosis” by the Infectious Diseases Society of America [26]. These recommendations were applied and led to the resolution of the illness in this patient.

## 4. Conclusion

This case of severe pulmonary blastomycosis in an immunocompetent young adult highlights the diagnostic challenges associated with this infection. The patient's history of exposure to organic material from his occupation as a gutter cleaner and his chronic e-cigarette use likely exacerbated the severity of the disease, underscoring the importance of lifestyle factors in the clinical assessment of respiratory infections. Early recognition and prompt diagnosis are essential to prevent disease dissemination and reduce complications. Given that blastomycosis is a rare disease and is not reportable in most states, many cases may remain undetected or be misdiagnosed. Future research should investigate the potential relationship between the severity of blastomycosis infection and e-cigarette usage. We also encourage future researchers to contribute toward the changing epidemiology and guideline-mediated treatment approaches for e-smokers with infections.

## Figures and Tables

**Table 1:** Investigations and their results.

Investigations	Results	Reference
Upper respiratory panel (influenza A, RSV, and COVID 19)	Negative	
*Immunoglobulins*		
IgA	165.5 mg/dL	> or = 18 years: 61–356 mg/dL [10]
IgM	107.1 mg/dL	> or = 18 years: 37–286 mg/dL [11]
IgG	910 mg/dL	> or = 18 years: 767–1590 mg/dL [12]

*Infectious disease serology*		
EBV IgG antibody	100 U/mL (high)	
CMV IgG antibody	Positive	
Coccidioides antibody screening	Negative	
Cryptococcus antigen	Negative	
Chlamydia IgG antibody	IgG < 1 : 64	
Mycoplasma IgG antibody	Positive	
TB PCR	Not detected	
HIV 1/2 antibodies	Nonreactive	

*Fungal infections*		
(1,3)-Beta-D-glucan	< 31 pg/mL	< 10–40 pg/mL [13]
*Blastomyces* antigen (urine)	Detected	
Histoplasma galactomannan antigen (urine)	Detected	

*Tissue histology*		
Right lung, upper lobe, and bronchoalveolar lavage	Rare budding yeast organisms present, extensive neutrophilic inflammation present	
Right lung, upper lobe, and transbronchial biopsy	Budding yeast present with associated acute and chronic inflammation; organizing pneumonia	
10-R lymph node and fine-needle aspiration biopsy	Budding yeast present	

*T cell subsets*		
CD3	95%	60%–85%
CD4	71%	30%–60%
CD8	10%	20%–40%
CD4:CD8 ratio	7:1	
Lymphocytes (CD3+/CD4−/CD8−)	14%	

*Autoimmune markers*		
Myeloperoxidase antibodies IgG	Not elevated	
Serine protease 3 IgG	Not elevated	
ANCA IFA pattern	None detected	

*Renal function (creatinine)*		
On the day of AKI	3.13	0.8–1.3
Day of amphotericin initiation (4 days from AKI)	1.95	0.8–1.3
Day of discharge	1.01	0.8–1.3

*Liver function (AST/ALT)*		
Day of amphotericin initiation	46/47	< 48/< 61
Peak elevation (2 days from initiation)	177/160	< 48/< 61
Day of discharge	25/87	< 48/< 61

## Data Availability

Data are available on request due to privacy restrictions.
